# Effects of Hyperoxia on Aging Biomarkers: A Systematic Review

**DOI:** 10.3389/fragi.2021.783144

**Published:** 2022-01-03

**Authors:** Belay Tessema, Ulrich Sack, Zoya Serebrovska, Brigitte König, Egor Egorov

**Affiliations:** ^1^ Institute of Clinical Immunology, Faculty of Medicine, University of Leipzig, Leipzig, Germany; ^2^ Institute of Medical Microbiology and Epidemiology of Infectious Diseases, Faculty of Medicine, University of Leipzig, Leipzig, Germany; ^3^ Department of Medical Microbiology, College of Medicine and Health Sciences, University of Gondar, Gondar, Ethiopia; ^4^ Department of Hypoxic States Investigation, Bogomoletz Institute of Physiology of National Academy of Sciences of Ukraine, Kyiv, Ukraine; ^5^ Ipam Institute for Preventive and Anti-Aging Medicine, Berlin, Germany

**Keywords:** hyperoxia, aging, age-related diseases, aging biomarkers, effects

## Abstract

The effects of short-term hyperoxia on age-related diseases and aging biomarkers have been reported in animal and human experiments using different protocols; however, the findings of the studies remain conflicting. In this systematic review, we summarized the existing reports in the effects of short-term hyperoxia on age-related diseases, hypoxia-inducible factor 1α (HIF-1α), and other oxygen-sensitive transcription factors relevant to aging, telomere length, cellular senescence, and its side effects. This review was done as described in the Preferred Reporting Items for Systematic Reviews and Meta-Analyses (PRISMA) guideline. A systematic search was done in PubMed, Google Scholar, and Cochrane Library and from the references of selected articles to identify relevant studies until May 2021. Of the total 1,699 identified studies, 17 were included in this review. Most of the studies have shown significant effects of short-term hyperoxia on age-related diseases and aging biomarkers. The findings of the studies suggest the potential benefits of short-term hyperoxia in several clinical applications such as for patients undergoing stressful operations, restoration of cognitive function, and the treatment of severe traumatic brain injury. Short-term hyperoxia has significant effects in upregulation or downregulation of transcription factors relevant to aging such as HIF-1α, nuclear factor kappa-light-chain-enhancer of activated B-cells (NF-kB), and nuclear factor (erythroid-derived 2)-like 2 (NRF2) among others. Short-term hyperoxia also has significant effects to increase antioxidant enzymes, and increase telomere length and clearance of senescent cells. Some of the studies have also reported adverse consequences including mitochondrial DNA damage and nuclear cataract formation depending on the dose and duration of oxygen exposure. In conclusion, short-term hyperoxia could be a feasible treatment option to treat age-related disease and to slow aging because of its ability to increase antioxidant enzymes, significantly increase telomere length and clearance of senescent cells, and improve cognitive function, among others. The reported side effects of hyperoxia vary depending on the dose and duration of exposure. Therefore, it seems that additional studies for better understanding the beneficial effects of short-term hyperoxia and for minimizing side effects are necessary for optimal clinical application.

## Introduction

Aging can be characterized as impaired organ functions, increased vulnerability for diseases and death, and decreased physiological integrity. Aging can also cause increased risk of multiple coexisting diseases, impaired response to stress, changed response to treatment, increased risk of disability, and loss of personal power that has major psychological and social consequences (Colloca et al., 2020). This biological weakening associated to aging is considered to be the main predisposing factor for cancer, cardiovascular diseases, diabetes, and Alzheimer’s disease among others. At the cellular level, there are key hallmarks of the aging process such as genomic instability, telomere shortening, cellular senescence, epigenetic changes, mitochondrial dysfunction, decreased autophagy, decreased proteostasis, stem cell collapse, decontrolled nutrient-sensing, and changed intercellular communication (López-Otín et al., 2013).

Adaptive homeostasis is a result of specific and selective activation of intracellular signal-transduction pathways caused by extremely low and non-damaging levels of signaling agents such as reactive oxygen species (ROS) (Davies, 2016; Pomatto and Davies, 2018). Studies have suggested that the age-associated decline in adaptive homeostasis is a major risk factor for many age-associated diseases (Pomatto et al., 2017; Pomatto et al., 2018). Cellular adaptive homeostasis during the fluctuations in oxygen availability is maintained by definite, fast, and effective cellular mechanisms, mostly depending on the quick and crucial effect of the two transcription factors such as the hypoxia-inducible factor (HIF)-1α (HIF-1α) and nuclear factor (erythroid-derived 2)-like 2 (NRF2). NRF2 is stimulated by hypoxia and hyperoxia. Hypoxia can also simulate HIF-1α and other HIF family members such as HIF-1β, HIF-2, and HIF-3. The cellular response stimulated by HIF-1α and NRF2 is balanced by another transcription factor called nuclear factor kappa-light-chain-enhancer of activated B-cells (NF-kB) that also helps in defining the consequence of the response to oxygen change and to cellular damage (Fratantonio et al., 2018).

HIF-1α stimulates multiple genes participating in cell survival, probably to gain time to reset homeostatic mechanisms (Semenza, 2002). In humans and animals, HIF-1α modulated genes that are recognized to be participating in improving vascular biology, such as vascular endothelial growth factor (VEGF), platelet-derived growth factor (PDGF), and erythropoietin (Semenza, 2002). HIF-1α has long been recognized to participate in age-related diseases; for example, solid tumors show stabilization of HIF-1α to increase vascularization and growth under hypoxic conditions. More recently, numerous studies have indicated a more important role of HIF-1α as a direct modulator of aging. Studies have shown that HIF-1α significantly increases the life span of adult *Caenorhabditis elegans* (Cypser and Johnson, 2002; Pitt et al., 2014). In addition to modulating aging, the HIF-1α pathway is also interrelated with other aging pathways, such as mechanistic target of rapamycin (mTOR), insulin-like signaling, and dietary restriction (Pitt et al., 2014).

Hyperbaric oxygen therapy (HBOT) has been suggested for several conditions including age-associated diseases for more than 40 years (Ingle, 1990; Leach et al., 1998), and it has recently been promoted as an approach to slow aging. HBOT is a method of treatment in which patients breathe in oxygen through a head tent, mask or endotracheal tube inside a hyperbaric chamber. HBOT is usually administered at greater than one and less than three absolute atmosphere (ATA) and induces a state of higher pressure and hyperoxia that cause mechanical and physiologic effects (Marcinkowska et al., 2021).

Depending on the level of dissolved O_2_ and pressure, HBOT activated diverse innate repair mechanisms (Efrati and Ben-Jacob, 2014). However, the relation between O_2_ and pressure remains unknown. The therapeutic effect of HBOT is the result of increasing the partial pressure of oxygen in the tissues of the body. Furthermore, HBOT increases the oxygen-carrying ability of blood plasma more than its ability under normobaric conditions. Nowadays, HBOT is being used to treat CO poisoning, delayed radiation injuries, decompression sickness, as well as non-healing diabetic wounds and others (Buras, 2000). Furthermore, hyperoxia O_2_ preconditioning has been revealed to have hormetic effects on stress resistance and to increase the longevity of *C. elegans* (Cypser and Johnson, 2002) and house flies (Sohal et al., 1993). Although the mechanism by which hyperoxia increases longevity is unclear, it is probably associated to the fact that hyperoxia can induce ROS production (Rothfuss and Speit, 2002). Small amount of ROS could induce protective gene expression and help cells and tissues to manage several stressors more efficiently.

The intermittent hyperoxia administered repeatedly based on defined HBOT protocols can cause physiological changes that typically happen during hypoxia, the phenomenon named hyperoxic–hypoxic paradox (Cimino et al., 2012; Sunkari et al., 2015). Additionally, it was recently revealed that HBOT can induce cognitive enhancements in healthy aging adults through changes in cerebral blood flow (Shwe et al., 2021). On the cellular level, it was confirmed that HBOT can induce the expression of HIF-1α, VEGF, and sirtuin (SIRT), stem cell proliferation, mitochondrial biogenesis, angiogenesis, and neurogenesis (Hadanny and Efrati, 2020), increase telomere length, and decrease senescent cell concentration (Hachmo and Hadanny, 2020). These findings imply that HBOT has anti-aging effects.

There is an impressive number of publications dedicated to the therapeutic effect of HBOT in various pathologies and to improve the quality of life of healthy people. There are also reports of the positive effects of HBOT on parameters related to lifespan and its side effects. However, it remains unclear how HBOT affects the aging process. Prolonged hyperoxia also has the property of stimulating free radical oxidation and tissue damage. In this review, we analyzed the reports on the impact of HBOT on various markers of aging, life expectancy, and quality of life, and on the potential side effects of a short-term therapeutic regimen of hyperoxia.

## Methods

This systematic review was done based on the guideline of Preferred Reporting Items for Systematic Reviews and Meta-Analyses (PRISMA) in accordance with the published protocol (registration number = CRD42021265130) (Moher et al., 2009).

### Data Sources and Search Strategy

Electronic databases including PubMed, Google Scholar, and Cochrane Library were searched from the year 1946 to 2021 for relevant studies; last search was performed on May 11, 2021. The lists of references of included studies were manually searched for additional studies. The following search terms were used for comprehensive search of available relevant studies from the electronic databases: (1) PubMed: ((((((((((((Hyperoxia (MeSH Terms)) OR “Hyperbaric Oxygen" (MeSH Terms)) OR “Intermittent hyperoxia" (MeSH Terms)) OR “Hyperoxic-Hypoxic" (MeSH Terms)) OR Normoxia (MeSH Terms)) OR “Normobaric Oxygen" (MeSH Terms)) OR Normoxic (MeSH Terms)) OR Oxygen (MeSH Terms)) AND Aging (MeSH Terms))) OR Antiaging (MeSH Terms)) OR Aging parameters) OR Antiaging parameters (MeSH Terms). (2) Google Scholar: allintitle: Hyperoxia OR “Hyperbaric Oxygen” OR “Intermittent hyperoxia” OR “Hyperoxic-Hypoxic” OR Normoxia OR “Normobaric Oxygen” OR Normoxic OR Oxygen AND Aging OR “anti-aging” OR “aging parameters” OR “anti-aging parameters”. (3) Cochrane Library: Trials matching Hyperoxia OR Hyperbaric and aging in Record Title (Word variations have been searched).

### Study Selection and Eligibility Criteria

After removal of duplicates, a two-step approach was used to select articles. Firstly, titles and abstracts of all search results were screened for the relevant original article published in English. Secondly, full-text articles were downloaded from the selected studies and were reviewed on the following inclusion and exclusion criteria:

#### Inclusion Criteria

Studies that performed hyperbaric oxygen therapy (HBOT) or normobaric hyperoxic training (NHOT) with described protocol; experimental studies done in human, animal, or cell line models; and studies done to assess the effect of HBOT or NHOT on potential anti-aging hallmarks and/or biomarkers including genomic instability, telomere shortening, cellular senescence, epigenetic changes, mitochondrial dysfunction, decreased autophagy, decreased proteostasis, stem cell exhaustion, deregulated nutrient-sensing, altered intercellular communication, oxidative stress, and antioxidants were included in this systematic review.

#### Exclusion Criteria

Reports other than original article such as case studies, conference proceedings, case series, review articles, and dissertations or thesis; studies that used both oxygen and pharmacological interventions together; studies that performed both hyperoxia and exercises together; and articles published in a language other than English were excluded from the review.

### Data Extraction

Data extraction was performed using structured form on Excel. The name of authors, year of publication, country where primary studies were conducted, study design, hyperoxia protocol, aging biomarkers investigated, results, author’s conclusions, oxygen toxicity, and data for quality assessment among other information associated with the review question were collected. Two of the authors (BT and BK) independently reviewed titles, abstracts, full texts, and extracted data. Consistencies of selected studies and extracted information obtained from the two authors were compared. The discrepancies were resolved after through discussion and agreed on the information. The review process and results from the systematic literature search are summarized in figure while the extracted data from the selected studies are summarized in tables.

### Data Analysis

In this review, the methodological variations hindered merging quantitative data from the individual studies in a meta-analysis. Therefore, we present data on the primary outcomes of hyperoxia intervention groups by using a qualitative comparison with the control group in a systematic review.

## Results

### Search Results and Included Studies Characteristics

**FIGURE 1:**
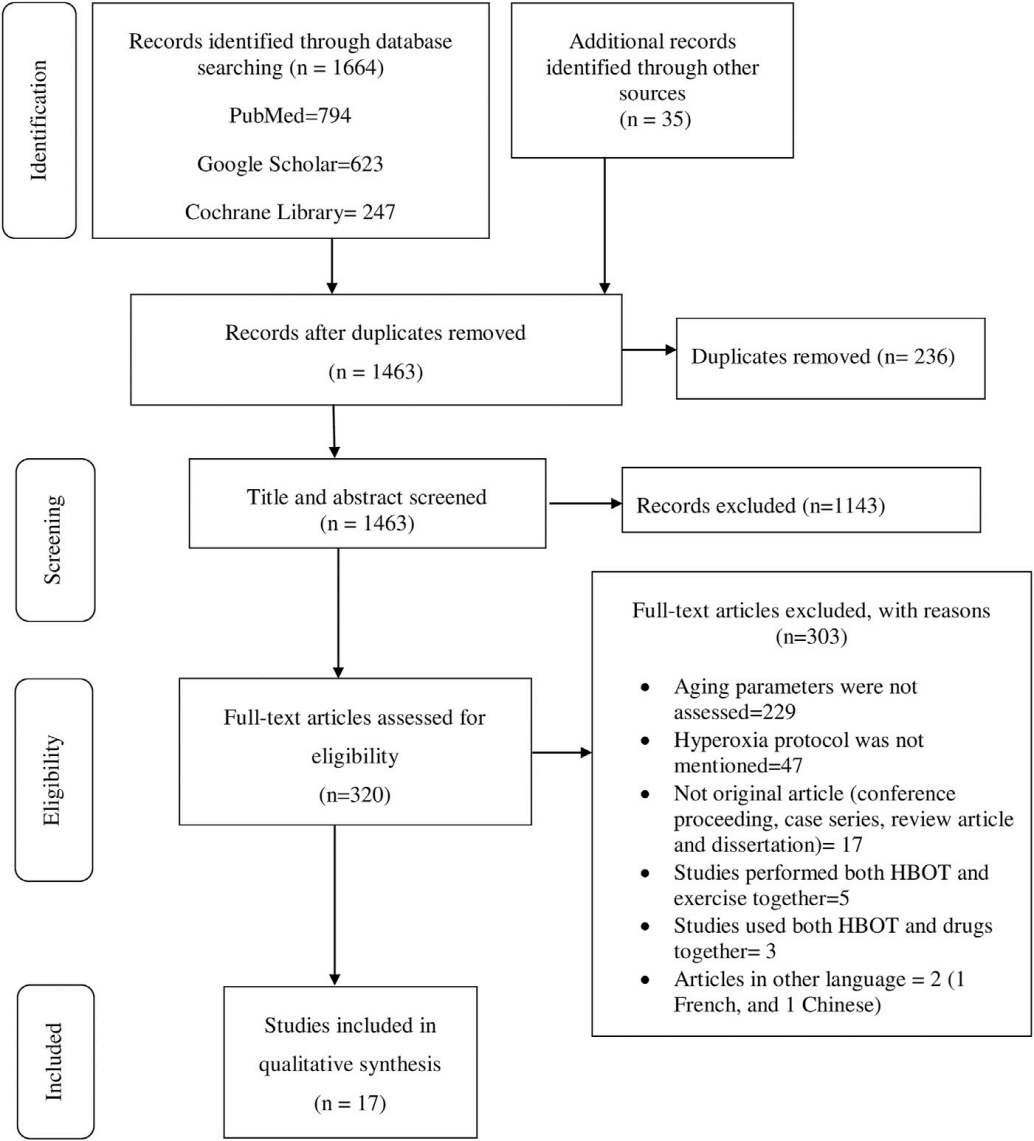
PRISMA flow diagram shows the search strategy and screening of eligible studies at different levels of the review process (Moher et al., 2009).

The initial search generated 1,664 articles (794 from PubMed, 623 from Google Scholar, and 247 from Cochrane Library) and hand searching of reference lists of included studies found 35 additional articles; most of these were excluded for the reasons mentioned in Figure 1. Finally, 17 articles were selected for data extraction and included in this systematic review. Of the 17 articles, nine studies were performed on humans (Table 1, Supplementary Table S1), two studies were done on cell line models (Table 2, Supplementary Table S2), three studies on rats (Table 3, Supplementary Table S3), and three studies were conducted on insects and worms (Table 4, Supplementary Table S4). Nine studies were randomized controlled clinical trials (RCT), six studies were non-randomized clinical trials, and the remaining two studies were uncontrolled clinical trials. The age groups of study participants in the studies included in this review range from 18 to 80 years old for humans (Table 1), 4 days to 14 months for rats (Table 3), and 3–59 days old for insects and worms (Table 4).

**TABLE 1:** The effect of hyperoxia on aging markers in human.

Author, year	Condition	Age in years	Hyperoxia protocol	Aging markers	Results	Conclusion	Safety issues (oxygen toxicity)
Amir et al. (2020)	Healthy	Adults (>64)	100% O_2_ at 2ATA for 90 min with 5-min air breaks every 20 min	CBF	Increased	HBOT induces cognitive enhancements in healthy aging adults *via* mechanisms involving regional changes in CBF.	Mild middle ear barotrauma, visual acuity changes, far sight acuity deterioration
Fratantonio et al. (2021)	Healthy	Adults (mean, 21)	30% O_2_ (MH) for 1 h	HIF-1α	Activated	The return to normoxia after MH is sensed as a hypoxic trigger characterized by HIF-1 activation. On the contrary, HH and VHH induce a shift toward an oxidative stress response, characterized by NRF2 and NF- kB activation	Not reported
				NRF2	Activated		
				NF- kB	Not activated		
				GSH	Not activated		
				MMP-9 and MMP-2	Activated (MMP-9)		
Fratantonio et al. (2021)	Healthy	Adults (mean, 21)	100% O_2_ (HH) for 1 h	HIF-1α	Activated		
				NRF2	Activated		
				NF- kB	Activated		
				GSH	Activated		
				MMP-9 and MMP-2	Activated		
Fratantonio et al. (2021)	Healthy	Adults (mean, 21)	140% O_2_ (VHH) for 1 h	HIF-1α	Not activated		
				NRF2	Activated		
				NF- kB	Activated		
				GSH	Activated		
				MMP-9 and MMP-2	Activated (MMP-9)		
García-de-la-Asunción et al. (2011)	Colon cancer	Adults (18–80)	30 or 80% O_2_ throughout surgery	MDA levels	Lower in the 80% O_2_ group than in the 30% O_2_ group	An increase in oxidative stress marker levels in blood and colonic mucosa occur when 30% O_2_ is used, possibly through an increase in XO enzymatic activity in the colonic mucosa. The 80% O_2_ prevented oxidative stress, with a reduction of lipid peroxidation and glutathione oxidation; this may be due to decreases in XO enzymatic activity and XO/(XO + XDH) ratio in the colonic mucosa	Authors could not find respiratory complications in their patients during the study period. Administration of 80% O_2_ during surgery and 2 h after surgery did not worsen pulmonary function or cause atelectasis
				GSSG	Lower in the 80% O_2_ group than in the 30% O_2_ group		
				XDH	XDH was higher, but XO/(XO + XDH) ratio was lower in the 80% O_2_ group than in the 30% O_2_ group		
				XO	XO and XO/(XO + XDH) ratio were lower in the 80% O_2_ group than in the 30% O_2_ group		
Hachmo and Hadanny, (2020)	Healthy	Adults (>64)	100% O_2_ at 2ATA for 90 min with 5-min air breaks every 20 min	Telomere length	Telomeres length of T helper, T cytotoxic, natural killer, and B cells increased	The study indicates that HBOT may induce significant senolytic effects including significantly increasing telomere length and clearance of senescent cells in the aging populations	Not reported
				Senescent cells	Decrease in the number of senescent T helpers and T-cytotoxic senescent cells		
				HIF-1α	HIF-1 α levels increased		
Karu et al. (2007)	Coronary artery diseases	Adults (mean age >60)	>96% O_2_ for 120 min before cardioplegia	Troponin I	Did not differ between the groups	Exposure to >96% oxygen before cardioplegia did not attenuate ischemia–reperfusion injury of the heart in patients undergoing coronary artery bypass grafting. The only potentially beneficial effect observed was the decreased transmyocardial release of interleukin-6	Not reported
				CK-MB	Did not differ between the groups		
				Lactate	Did not differ between the groups		
				GSH	More oxidized GSH was released in the hyperoxia group		
				Il-6	Decreased release of IL-6		
Karu et al. (2015)	Coronary artery	Adults (>54)	100% O_2_ for 60 min	MTRNR2L2 and MTRNR2L8 genes	MTRNR2L2 and MTRNR2L8 upregulated, and a “cell survival” network was activated	Administration of 100% oxygen for 1 h changes gene expression in the myocardium of the patients with coronary artery disease and may enhance cell survival capability	Not reported
Keramidas et al. (2011)	Healthy	Adults (mean age 25.5)	100% O_2_ for 2 h	EPO	EPO concentration was significantly lower in hyperbaric than in the normobaric condition	The increased O_2_ tension suppresses the production of EPO in 3–5 h after the hyperoxic breathing intervention	Not reported
Ottolenghi et al. (2019)	Abdominal surgery	Adults (18+)	80% O_2_ during the surgery and until 2 h after the end of surgery	Hydroperoxides	Hydroperoxides did not highlight any differences between the two groups nor within the same group, with respect to the baseline value	MDA, the main end product of the peroxidation of polyunsaturated fatty acids directly influenced by O_2_, may represent the best marker to assess the pro-oxidant/antioxidant equilibrium after surgery	Unbalanced pro-oxidant/antioxidant equilibrium
				Antioxidants	Antioxidant defense lower, in the 80% O_2_ group with respect to both the 40% O_2_ group and the baseline values		
				NOx	NOx was higher in the 80% O_2_ group than the 40% O_2_ group at 2 h after surgery		
				MDA	The MDA concentration was higher 24 h after surgery in the 80% O_2_ group with respect to both the 40% O_2_ group and the baseline values		
				HbSSG	HbSSG in red blood cells was higher in the 80% O_2_ group at the end of the surgery		
Rockswold et al. (2010)	Severe traumatic brain injury	Adults (average, 35)	100% O_2_ for 60 min at 1.5 ATA (HBO_2_) or 100% O_2_ for 3 h at 1.0 ATA (NBH)	CBF	Hyperbaric O_2_ significantly increased CBF for 6 h	Hyperbaric O_2_ has a more robust posttreatment effect than NBH on oxidative cerebral metabolism	No signs of pulmonary or cerebral O_2_ toxicity
				CSF lactate, glucose, pyruvate, and glycerol level	CSF lactate concentrations decreased in both the HBO_2_ and NBH groups. The dialysate lactate levels in HBO_2_ decreased. Microdialysis lactate/pyruvate (L/P) ratios decreased in both HBO_2_ and NBH groups. No increase in microdialysate glycerol		
				CSF F2-isoprostane	No increase in the CSF F2-isoprostane levels		
				BAL fluid IL–8 and IL-6	No increase in BAL inflammatory markers, IL-6, and IL-8		

MH, Medium hyperoxia; HH, High hyperoxia; VHH, Very high hyperoxia; CBF, Cerebral blood flow: HIF-1 α, Hypoxia-inducible factor-1 α; NRF2, Nuclear factor (erythroid-derived 2)-like 2; NF- kB, Nuclear Factor kappa-light-chain-enhancer of activated B cells; GSH, Glutathione; MMP-9/2, Matrix metallopeptidase- 9/2; MDA, malondialdehyde; GSSG, oxidized glutathione; XDH, xanthine dehydrogenase; XO, xanthine oxidase; CK-MB, creatine kinase-MB; IL-6/8, interleukin-6/8; EPO, Erythropoietin; NOx, nitrates and nitrites; HbSSG, glutathionyl hemoglobin.

**TABLE 2:** The effect of hyperoxia on aging markers in cell lines.

Author and year	Cell line	Hyperoxia protocol	Aging markers	Results	Conclusion	Safety issues (oxygen toxicity)
Godman et al. (2010)	HMEC-1	100% O_2_ at 2.4 ATA for 1 h	Antioxidant gene expression in Nrf2, Integrin, and ERK/MAPK pathways	The HSPA1A, HMOX1, and MT1X genes were upregulated, and collectively can provide protection from metabolic, proteotoxic, and oxidative forms of stress. ERK/MAPK signaling, including the activation of a number of immediate early genes can potentially influence apoptotic signaling. Endothelial cell viability in the HBO-treated cultures was significantly increased	The data indicate that hyperbaric oxygen can induce protection against oxidative insults in endothelial cells and may provide an easily administered hormetic treatment to help promote healthy aging	HBO is a relatively low-risk procedure that could be effectively applied as a broader preventative regimen to reduce the effects of aging
Pomatto et al. (2019)	MEFs	40% O_2_ for 2 weeks	Nrf2 signal transduction pathway	Hyperoxia increased baseline levels of Nrf2 and multiple transcriptional targets (20S Proteasome, Immunoproteasome, Lon protease, NQO1, and HO-1)	Changes the balance of Nrf2, Bach1, and c-Myc levels may account for dysregulation of stress responses and adaptive homeostasis during chronic hyperoxia and in aging	Not reported
			Nrf2 inhibitors (Bach1 and c-Myc)	Bach1 and c-Myc were strongly elevated by hyperoxia and appeared to exert a ceiling on Nrf2 signaling. Bach1 and c-Myc also increase during aging and may thus be the mechanism by which adaptive homeostasis is compromised with age		
			Cellular ability to adapt to signaling levels (1.0 μM) of H_2_O_4_	Hyperoxia resulted in loss of cellular ability to adapt to signaling levels (1.0 μM) of H_2_O_2_		

HMEC-1, Human microvascular endothelial cell line; MEFs, Mouse embryonic fibroblasts; HSPA1A, 70- kilodalton heat shock protein; HMOX1, heme oxygenase 1; MT1X, metallothionein 1X.

**TABLE 3:** The effect of hyperoxia on aging markers in rats.

Author and year	Condition	Age	Hyperoxia protocol	Aging markers	Results	Conclusion	Safety issues
Hosford, (2003)	Healthy	4–14 days	>95% O_2_ for 10 days	VEGF	mRNA levels of VEGF increased in normoxic animals, but hyperoxia suppressed this increase	Hyperoxic exposure decreased VEGF levels, and decreased VEGF receptors (VEGFR1 and VEGFR2) levels	Not reported
				VEGF receptors (VEGFR1 and EGFR2)	VEGFR1 and VEGFR2 mRNA increased in normoxic animals, but they were decreased by hyperoxia		
				HIF-2 α	mRNA levels of HIF-2 α increased in normoxic animals, but hyperoxia suppressed this increase		
Shwe et al. (2021)	NA	>20 weeks	100% O_2_ at 2 ATA for 80 min/day for 14 days	Synaptic plasticity [Markers: LTD, LTP, dendritic spine density, expression of synaptic protein (PSD 95)]	Synaptic plasticity was restored/improved	HBOT attenuated insulin resistance, cognitive impairment, hippocampal aging and pathologies. These findings suggest that HBOT restored insulin sensitivity, hippocampal functions, cognition in aging, and aging-obese models	Not reported
				Hippocampal insulin receptor function (marker: LTD)	Insulin receptor function was restored/improved		
				Hippocampal ROS level	ROS was decreased		
				DCX	Could not restore neurogenesis		
				Hippocampal autophagy (markers: p62 and LC3-II)	Hippocampal autophagy was restored		
				Microglia hyperactivation	Microglial hyperactivation was attenuated		
				Hippocampal apoptosis	Hippocampal apoptosis reversed back to normal		
				Aging marker: beta-secretase (BACE1)	BACE1 enzyme was reduced		
				Aging marker: telomere length	Telomere length was restored		
				Aging marker: SA-β-gal staining	The number of SA-β-gal-positive cells was decreased		
Zhang et al. (2010)	Healthy	2–14 months	60% O_2_ for 3 weeks	mtDNA damage	Increased	These data emphasize the importance of DNA repair enzymes and antioxidant enzymes as targets to promote DNA repair and reduce production of ROS.	Increasing the exposure of the lens to hyperoxia could lead to mtDNA damage and increase the risk of nuclear cataract formation
				mtBER enzymes	Increased		
				8-OHdG levels	Increased		

NM, not mentioned; VEGF, Vascular endothelial growth factor; LTD, Insulin-induced long-term depression; LTP, long-term potentiation; SA-β-gal, senescence-associated β falactosidase; DCFHDA, dichloro-hydrofluoresceindiacetate; DCX, neurogenesis; mtBER, mtDNA, base excision repair; mtDNA, mitochondrial DNA; LX-PCR, Long extension polymerase chain reaction; 8-OHdG, 8-hydroxy-20-deoxy-guanosine.

**TABLE 4:** The effect of hyperoxia on aging markers in insects and worms.

Author and year	Insect/worm	Condition	Age	Hyperoxia protocol	Aging markers	Results	Conclusion	Safety issues
Rebrin and Sohal, (2006)	*Drosophila melanogaster*	NA	9–59 days	100% O_2_ from 10 days old until death	GSH	Hyperoxia had no marked effect on GSH concentration in both WT and YW flies	Results indicated that hyperoxia (100% oxygen) neither reproduces nor accelerates the pattern of alterations in glutathione redox state and PrSSG content observed during aging under normoxic conditions	Not reported
					GSSG	Under hyperoxia, YW flies did not exhibit an increase in GSSG amount or a decline in GSH:GSSG ratio, whereas WT flies showed a decline in GSH:GSSG ratio only during the latter part of hyperoxia		
					PrSSG	In neither strain was there a progressive increase in PrSSG amount under hyperoxia		
Walker and Benzer, (2004)	*Drosophila melanogaster*	NA	3–4 days old	100% O_2_ was passed through the box at a constant rate (300 ml/min)	Degeneration of mitochondria	In hyperoxia condition, mitochondrial degeneration occurs rapidly within mitochondria of the flight muscle	Authors discovered a biomarker of oxidative damage to the mitochondria (swirls) within the flight muscle. Swirls may represent an early event in the deterioration of the mitochondrion	Degeneration of the mitochondria
Yanase and Ishii, (2008)	*Caenorhabditis elegans*	NA	5–15 days	90% O_2_ for 3 h per day for 10 days	Mitochondrial superoxide radical (O_2_-) levels	The O_2_- levels in age 1 strain significantly decreased after intermittent hyperoxia exposure	These data suggest that oxidative stress-induced hormesis is associated with a reduction in mitochondrial O_2_- production by activation of the antioxidant system *via* Ins/IGF-1 signaling pathway	Not reported

NA, not applicable; GSH, Glutathione; GSSG, glutathione disulfide; PrSSG, protein mixed disulfides; O_2_, superoxide; Ins/IGF-1, Insulin/Insulin-like growth factor-1.

Among nine studies done on human subjects, 5 studies were done on patients with age-related diseases: 2 studies on patients with coronary artery disease, one on colon cancer, one on patients undergoing abdominal surgery, and one on severe traumatic brain injury patients, and 4 studies were done on healthy individuals. This review assessed the effects of short-term hyperoxia on the following aspects of anti-aging: therapeutic effects of short-term hyperoxia on age-related diseases (6 studies); effects of short-term hyperoxia on HIF-1α, its targets, and other genes expression relevant to aging (4 studies); effect of short-term hyperopia on telomere length and cellular senescence (2 studies); and side effects of hyperoxia (7 studies).

According to the Cochrane tool for risk of bias assessment checklist (Higgins et al., 2011), the risk of incomplete outcome data bias (short term), incomplete outcome data bias (long term), and selective reporting bias were low in all studies. However, eight studies had unclear risk of allocation concealment bias, blinding of outcome assessment bias, and blinding of outcome assessment bias (all case mortality). Six and 2 studies had high and unclear risks of random sequence generation bias, respectively. Similarly, one and 7 studies had high and unclear risks of blinding of participants and personnel bias, respectively.

### Therapeutic Effects of Short-Term Hyperoxia on Age-Related Diseases

Several studies have reported the therapeutic effects of hyperoxia on age-related diseases (Tables 1, 3 and Supplementary Tables S1, S3). A RCT study by Amir et al. evaluated the effect of HBOT on cognitive performance in healthy aging adults (age >64 years) (Amir et al., 2020). The protocol consists of 60 daily sessions, 5 sessions per week in 3 months. Every session consisted of inhalation of 100% O_2_ for 90 min at 2ATA with air breaks (5 min) every 20 min. HBOT was demonstrated to induce cognitive enhancements in healthy aging adults through mechanisms increasing in cerebral blood flow (CBF). The main enhancements comprise information processing speed, executive functions, and attention, which usually weaken with aging. In another RCT study conducted by Shwe et al., at week 20, Wistar rats were given either sham treatment (1ATA, 80 L/min, 80 min/day) or hyperbaric O_2_ treatment (2ATA, pure O_2_, 250 L/min, 80 min/day) for 14 days (Shwe et al., 2021). Hyperbaric treatment reduced insulin resistance, cognitive impairment, hippocampal aging, and pathologies in rats. The results of these studies indicate that hyperoxia treatment could restore insulin sensitivity, hippocampal performance, and cognition during aging.

García-de-la-Asunción et al. investigated whether 80% fraction of inspired oxygen (FiO_2_) inhalation throughout surgery reduces xanthine oxidase (XO) action in colonic mucosa as a likely mechanism of decreasing oxidative stress during colon surgery of colon cancer patients (García-de-la-Asunción et al., 2011). Authors demonstrated that malondialdehyde (MDA) and oxidized glutathione (GSSG) levels measured in blood plasma as well as MDA measured in colonic mucosa were smaller in the 80% FiO_2_ group than in the 30% FiO_2_ group. Otherwise, the XO/(XO + xanthine dehydrogenase (XDH)) ratio and the enzymatic activity of XO in colonic mucosa were less in the 80% FiO_2_ group than in the 30% FiO_2_ group. Authors concluded that the main source of ROS in patients during colon surgery may be XO. Inhaling 80% oxygen during colon surgery raises arterial partial pressure, and this treatment was related with decreased XO activity and intensities of oxidative stress in colonic mucosa.

A study conducted by Karu et al. assumed that hyperoxia (>96% O_2_, an average of 120 min) and, beforehand, cardioplegia could defend the myocardium against necrosis and stunning resulting from ischemia–reperfusion (Karu et al., 2007). The results of the study showed that hyperoxic pre-treatment did not reduce ischemia–reperfusion damage of the heart in patients during coronary artery bypass grafting; however, hyperoxia eliminated the myocardial production of interleukin-6 during 20 min of reperfusion. Similarly, Karu and his colleagues evaluated gene expression level after hyperoxic exposure (>96% O_2_ for 60 min) to examine the protective phenotype that can be caused by hyperoxia in patients before coronary artery bypass grafting (Karu et al., 2015). Exposure to >96% O_2_ significantly altered the expression of twenty different genes, together with upregulation of two different humanins, namely, MTRNR2L2 and MTRNR2L8, and stimulated a cell survival network, which may increase cell survival ability. Rockswold et al. compared the treatment effects of hyperbaric O_2_ (1.5 ATA for 60 min) and normobaric hyperoxia (100% O_2_ at 1 ATA for 3 h) on severe traumatic brain injury (TBI) (Rockswold et al., 2010). Authors reported that hyperbaric O_2_ has a stronger posttreatment result than normobaric hyperoxia on oxidative cerebral metabolism associated to its ability to yield a brain tissue P O_2_ ≥ 200 mm Hg.

### Effects of Short-Term Hyperoxia on HIF-1α, Its Targets, and Other Oxygen-Sensitive Transcription Factors Relevant to Aging

In the process of aging, HIF-1α causes a defect in mitochondrial growth and division, which damages cellular processes dependent on energy, such as cell and tissue repair (Yuan et al., 2016). This leads the accumulation of reactive oxygen species, oxidation of lipids and proteins, and mitochondrial DNA mutations, which hasten the aging process by causing a decline in cellular energetics, the cellular redox condition, cell signaling, and calcium homeostasis (Yuan et al., 2016). Consequently, the mechanisms regulating HIF-1α have been associated with the prevention of the pathogenesis of many aging-related chronic diseases and premature cellular senescence. Several studies have shown that exposure to short-term hyperoxia at different concentrations and different lengths of time could upregulate or downregulate oxygen-sensitive transcription factors relevant to aging (Tables 1–3, Supplementary Tables S1–S3).

Fratantonio et al. investigated the effect of hyperoxia on transcription factors in human PBMCs isolated from healthy individuals after 1 hour inhalation of mild, high, and very high hyperoxia, corresponding to 30%, 100%, and 140% oxygen, respectively (Fratantonio et al., 2021). The exposure to 30% O_2_ was perceived by PBMCs as a hypoxic stress and characterized by the activation of NRF2 and HIF-1α, but not NF-kB. Conversely, exposure to 100% or 140% O_2_ was related to a rise in oxidative stress that causes the activation of NRF2 and NF-kB. Moreover, HIF-1α activation was absent after exposure to 140% O_2_. The intracellular levels of GSH and the levels of MMP-9 in plasma reflected the activation of NRF2 and HIF-1α, respectively.

Godman et al. assessed the possible beneficial effects of hyperbaric O_2_ as a mild hormetic stress on human microvascular endothelial cell line-1 (HMEC-1) (Godman et al., 2010). The HMEC-1 cells were grown and treated with HBO protocol (100% O_2_ for 1 h at 2.4 atm) in CO_2_-independent media. The study indicated an upregulation of immediate early genes and cytoprotective and antioxidant genes. This upregulation coincided with a higher resistance to a lethal oxidative stress. That may contribute to increase healthy aging.

Pomatto et al. investigated Nrf2 signaling in mouse embryonic fibroblast (MEF) cell lines grown under hyperoxic conditions (40% O_2_), as a model of accelerated aging (Pomatto et al., 2019). The findings of the study revealed that hyperoxia raised the initial amount of Nrf2 and many transcriptional targets including Immunoproteasome, 20S Proteasome, NQO1, Lon protease, and HO-1, but caused the loss of cellular capacity to adapt to signaling levels of H_2_O_2_. The levels of 2 main Nrf2 inhibitors, c-Myc and Bach1, were strongly raised by hyperoxia and seemed to exert a ceiling on Nrf2 signaling. The c-Myc and Bach1 also increase during aging. Therefore, these may be the mechanism through which adaptive homeostasis is affected during aging.

A study by Hosford et al. showed that hyperoxia exposure (>95% O_2_, days 4–14) halts lung alveolarization in rats and may do so *via* reduction of the VEGF signaling system (Hosford, 2003). The mRNA amount of HIF-2a and VEGF in lung tissue increased between day 4 and 14 in normoxic animals; however, hyperoxia inhibited these rises. Amounts of HIF-2α and VEGF mRNA were interrelated in the air but not the hyperoxia oxygen-treated group, signifying that the low amounts of HIF-2α detected at high oxygen are not inducing VEGF expression. The expression of mRNA of VEGFR1 and VEGFR2 mRNA also increased in air-exposed animals, and these, too, were considerably decreased by hyperoxia between day 9 and day 12, respectively. Receptor protein amounts did not rise with development; however, oxygen did reduce protein to less than air levels. Authors concluded that hyperoxic reduction of VEGF signaling from days 9 to 14 may be one of the mechanisms through which alveolarization is arrested.

### Effects of Short-Term Hyperoxia on Telomere Length and Cellular Senescence

Studies have investigated the effects of hyperoxia on some of the key hallmarks of aging such as telomere length shortening and cellular senescence (Tables 1, 3 and Supplementary Tables S1, S3). Hachmo et al. assessed whether hyperoxia (100% O_2_ at 2ATA for 90 min) changes the telomere length and senescent cell number in normal, non-pathological, aging adults (aged ≥64) (Hachmo and Hadanny, 2020). The study showed that the telomere length of T helper, T cytotoxic, natural killer, and B cells was restored markedly by more than 20% after hyperoxia treatment. The highest change was detected in B-cell count, which increased markedly at the 30th session, 60th session, and after hyperoxia treatment. The number of senescent T helpers and T-cytotoxic senescent cells were markedly reduced after hyperoxia treatment. The authors concluded that hyperoxia treatment may stimulate marked senolytic effects including markedly rising telomere length and clearance of senescent cells in the aging individuals. Similarly, another study conducted by Shwe et al. investigated the effect of hyperoxia (at 2ATA, pure O_2_, 250 L/min, 80 min/day) for 14 days on telomere length in hippocampus in rats (Shwe et al., 2021). The study showed that HBOT restored telomere length in hippocampus.

### Side Effects (Toxicity) of Short-Term Hyperoxia

Studies have assessed the potential side effects of hyperoxia at different oxygen doses and exposure times (Tables 1–4, Supplementary Tables S1–S4). Some of the studies have shown that exposure to hyperoxia may cause some side effects (Walker and Benzer, 2004; Zhang et al., 2010; Ottolenghi et al., 2019; Amir et al., 2020). A randomized controlled clinical trial study by Amir et al. evaluated the side effects of HBOT (100% O_2_ at 2ATA for 90 min) (Amir et al., 2020). In the oxygen treatment group, four participants (13.3%) had mild middle ear barotrauma compared to none in the control group. Sixty-two percent of the participants without intraocular lens implantation had visual acuity changes in the oxygen treatment group, compared to 37% in the control group. In the oxygen treatment group, nine patients (31.0%) experienced far sight acuity weakening while six patients (20.6%) experienced improvement in their far sight acuity.

Zhang and his colleagues compared the effects of hyperoxic (60% O_2_), hypoxic (11% O_2_), and normoxic (21% O_2_) exposures of rats’ eyes for 3 weeks on the mitochondrial DNA (mtDNA) damage, gene expression of mtDNA base excision repair (mtBER) enzymes, and 8-hydroxy-2′ -deoxyguanosine (8-OHdG) level in lens (Zhang et al., 2010). The study showed the damage of mtDNA, the expression of mtBER enzymes, and the level of 8-OHdG in lens raised following hyperoxia, which is likely related with oxidative stress. In the O_2_ treatment group, nuclear cataract was developed quickly at 14-month-old rats while lens remained transparent in the control group. Similarly, a study by Walker et al. revealed a prominent initial pattern of degeneration of the mitochondria in *Drosophila* flight muscle under hyperoxia (100% O_2_) (Walker and Benzer, 2004). Similarly, malondialdehyde (MDA), the main finale product of the peroxidation of polyunsaturated fatty acids, was directly affected by exposure to 80% Fi O_2_ during and after general anesthesia (Ottolenghi et al., 2019).

In contrast, some other studies have demonstrated that exposure to hyperoxia do not cause potential side effects (Godman et al., 2010; Rockswold et al., 2010; García-de-la-Asunción et al., 2011). Rockswold et al. compared hyperbaric oxygen (1.5 ATA for 60 min) and normobaric hyperoxia (1 ATA for 3 h) oxygen toxicity in severe traumatic brain injury (TBI) patients (Rockswold et al., 2010). However, there was no rise in the bronchoalveolar lavage (BAL) inflammatory markers (IL-6 and IL-8), microdialysate glycerol, and CSF F2-isoprostane levels, which were used to assess possible O_2_ toxicity. Godman and his colleagues also demonstrated an upregulation of cytoprotective, antioxidant, and immediate early genes in human endothelial cells following a hyperbaric oxygen exposure to 100% O_2_ at 2.4 atm for 1 h (Godman et al., 2010). These increases correlated with a higher level of resistance to a lethal oxidative stress. Similarly, García-de-la-Asunción et al. reported that administration of 80% O_2_ during colon surgery and 2 h after surgery did not worsen pulmonary function or cause atelectasis (García-de-la-Asunción et al., 2011). In conclusion, studies have reported varying degrees of side effects of hyperoxia from no side effect to serious side effects depending on the dose and duration of oxygen exposure in the protocol of the studies.

## Discussion

### Therapeutic Effects of Hyperoxia on Age-Related Diseases

Age-related diseases are broadly categorized as cardiovascular diseases, chronic respiratory diseases, communicable, maternal, neonatal, and nutritional diseases, diabetes and kidney diseases, digestive diseases, injuries, neoplasms, neurological disorders, sense organ diseases, skin and subcutaneous diseases, and other non-communicable diseases (Chang et al., 2019). One of the major results of this systematic review is that hyperoxia has significant therapeutic effects on some of these age-related diseases.

Hyperoxia can cause marked enhancements in cognitive function in healthy adults *via* mechanisms involving regional changes in CBF (Amir et al., 2020; Balasubramanian et al., 2021). The major enhanced domains are attention, information processing speed, and executive function in addition to global cognitive performance. These domains are known to gradually decline within the process of normal aging and play a crucial role in the daily functioning of the elderly (Harada et al., 2013). Other studies have also shown that a single O_2_ exposure can increase the cognitive functions including verbal function and visuospatial function *via* increased brain activation (Sohn et al., 2005; Chung et al., 2006). Similarly, HBOT restored cognitive function in both d-gal-induced aging and combined model of aging and obesity by reducing hippocampal pathologies (Shwe et al., 2021). Interestingly, HBOT attenuated brain aging and inflammation, as indicated by the decreased BACE level, increased relative telomere to single gene copy AT 1 receptor (T/S) ratio, decreased number of SA-β-gal positive cells, and decreased microglial hyper-activation.

HBOT (different ATA) could also restore memory in brain injury and D-galactose models, and the anti-aging and anti-inflammation effects of HBOT may reduce ROS production in D-gal-induced aging mice (Chen et al., 2016; Chen et al., 2017). HBOT attenuated D-gal-induced cognitive impairment by reducing oxidative stress in hippocampus *via* the increase of superoxide dismutase, glutathione peroxidase, and catalase levels in hippocampus (Chen et al., 2016). Hence, it was speculated that HBOT attenuates aging and inflammatory process in the brain and consequently decreases ROS production, which leads to decreased brain pathologies, resulting in restoration of cognitive function (Amir et al., 2020).

A reduction of MDA and GSSG amounts in the blood plasma and in the colonic mucosa of patients who inhaled 80% Fi O_2_ during colon surgery was reported (García-de-la-Asunción et al., 2011). MDA is an end product of fatty acid peroxidation in cell membranes and a classical marker of oxidative stress in tissues and blood (Wong et al., 1987). Another study also demonstrated a decrease in MDA blood plasma concentration and other oxidative stress marker levels such as GSSG in the blood of patients undergoing colon surgery with supplemental O_2_ (80% Fi O_2_) (García de la Asunción et al., 2007). Therefore, administration of 80% Fi O_2_ in patients undergoing colon surgery may protect them from the peroxidation of fatty acids in the colonic mucosa by oxidative stress and can be helpful in the decrease of surgical wound infections, duration of hospitalization, and return of bowel function (García de la Asunción et al., 2007).

Pretreatment with hyperoxia almost eliminated the postischemic release of IL-6 from the heart during reperfusion (Karu et al., 2007). This is in line with the previous reports (Wan et al., 1996; Colagrande et al., 2006). This diminished level of IL-6 shows a reduced myocardial injury, similar to a study that examined other cardioprotective strategies (Colagrande et al., 2006). However, the protective effect of hyperoxia on myocardial infarction or function was not detected. IL-6 levels during reperfusion have been demonstrated to be associated with the severity of injury, as assessed by left ventricular wall motion abnormalities (Hennein et al., 1994) and the negative inotropic effect (Finkel et al., 1992). Moreover, blockade of proinflammatory cytokines has been described to decrease neutrophil chemotaxis and sequestration and to reduce ischemia–reperfusion injury (Tassiopoulos et al., 1998).

A simple and broadly used therapeutic intervention (>96% O_2_ for 1 h) changes the gene expression profile in the myocardium of patients with coronary artery problem (Karu et al., 2015). Along with others, two distinct humanins were upregulated and survival-related genes and network were stimulated. The cytoprotective result of humanins has been recognized in several stress situations, such as oxidative stress (Yen et al., 2013). Hyperoxia treatment has also been demonstrated to decrease apoptotic cell death stimulated by ischemic insult increased Bcl-2/Bax ratio (Foadoddini et al., 2011). As part of cytoprotective effects, humanins can decrease cytochrome c release and decrease apoptosis as it has been demonstrated that humanin peptides block Bax relation with isolated mitochondria (Guo et al., 2003).

A more serious posttreatment consequence was also observed in patients with severe TBI after hyperbaric O_2_ treatment than normobaric O_2_ (Rockswold et al., 2010). Increased tissue levels of oxygen, positively influencing the binding of oxygen in mitochondrial redox enzyme systems, seemed to improve mitochondrial function. This improved function results in the more efficient use of baseline amounts of O_2_ after hyperbaric O_2_ treatment (Rockswold et al., 2001; Daugherty et al., 2004). Therefore, although the treatment effect of hyperbaric O_2_ in the early hours after TBI is important, it is not limited to that time period. It appears to be effective in improving oxidative cerebral metabolism during a much more prolonged period, which lasts for days (Rockswold et al., 2001).

### Effects of Hyperoxia on Oxygen-Sensitive Transcription Factors Relevant to Aging

Inhalation of 30% O_2_ for 1 h followed by a return to normoxia induced a significant activation of HIF-1α (Fratantonio et al., 2021). The activation of NRF2 showed the same trend; on the other hand, NF-kB was not affected during all the experimental times. These activation levels show that 30% oxygen stimulated a clean normobaric oxygen paradox (NOP) described by a mild but continued antioxidant response and a sharp, temporary activation of HIF-1α in the absence of a NF-kB response (Sies et al., 2017). In contrast to 30% O_2_, 100% oxygen induced a significant activation of NF-kB. In this circumstance, the whole response can be defined as a combination of oxidative stress response with a NOP. Inhalation of 140% O_2_ was related with marked oxidative stress. NOP is no longer evident and HIF-1α remains at the baseline levels; on the other hand, both NRF2 and NF-kB are significantly activated. Moreover, HIF-1α activation related with both 100% O_2_ and 30% was accompanied by a rise in EPO amounts of about 10%. This rise overlaps with the rise in circulating EPO after a pulsed hyperoxia already described by the studies following exposure to 100% and 50% O_2_ (Ciccarella et al., 2011).

Similarly, repetitive HBOT exposures increased HIF-1α expression and the HIF-1α levels gradually decreased towards normalization (Hachmo and Hadanny, 2020). Intermittent hyperoxic exposures induce many of the physiological responses that occur during hypoxia (Cimino et al., 2012). HBOT increases the release of HIFs and their stability as well as activity (Sunkari et al., 2015). In turn, HIF-1α induces mitochondria biogenesis, cellular cascade including vascular endothelial growth factor and angiogenesis induction, stem cell mobilization, and SIRT1 increased activity (Hadanny and Efrati, 2020). The HBOT can alter gene expression in a number of cellular pathways, such as the ERK/MAPK pathways, Integrin, and Nrf2 (Godman et al., 2010). These pathways comprise several genes that are crucial in cellular defense. HBO-treated cells are protected from lethal oxidative stress; this finding supports the functional significance of these gene expression changes.

Hyperoxia has potential benefits in several clinical applications related to age-related diseases. Some of the clinical applications include a pre-conditioning hyperoxia treatment, which may be an attractive choice for patients about to undergo mostly stressful operations, such as open-heart surgeries. Hyperoxia pretreatment has the capacity to protect humans from acute ischemia during coronary artery bypass grafting processes using cardiopulmonary bypass (Alex et al., 2005; Yogaratnam et al., 2006). Hyperoxia could also be considered as a possible treatment option to slow aging due to its capacity to raise endogenous antioxidant enzymes that decrease ROS-induced cellular impairment. Analysis of the gene array data also showed that hyperoxia can stimulate proliferation of HMEC-1 cells through the activation of growth-regulatory genes. Endothelial cell proliferation is a vital constituent of angiogenesis and wound healing, both of which decrease with age (Godman et al., 2010).

The role of Nrf2 signal transduction pathway in adaptive responses to oxidative stress is well known (Ma, 2013); however, its age-dependent decrease is not yet well understood. Homeostatic adaptive response is the temporary increase of stress-protective enzymes production and the activation of damage elimination and repair machinery necessary for clearance of dysfunctional enzymes, organelles, and lipids. These adaptive responses help to avert the accumulation of impaired cellular components, and to increase the probabilities of existence and strength in response to toxic conditions (Davies, 2016). As the main inducer of metabolic enzymes and multiple phase II detoxifications, temporary Nrf2 transcriptional activation is the cause for starting the adaptive homeostatic response to oxidants including H_2_O_2_. Nrf2 and its multiple downstream targets can be robustly increased at physiologically relevant conditions in response to an external stimulus (Pomatto et al., 2019). A basal increase in Nrf2 levels was reported in chronic diseases such as multiple forms of cancer (Moon and Giaccia, 2015) and with age (Pomatto et al., 2018). This shows a new baseline that allows organisms, tissues, or cells to cope with an age-associated rise in chronic oxidative stress generated, but the older organism was unable to further adapt or adjust the Nrf2 levels (Pomatto et al., 2019).

A study by Hosford et al. further increased the current body of knowledge to embrace the effects of hyperoxia on VEGFR1, VEGFR2, and HIF-2α (Hosford, 2003). The mRNA expression in VEGF rises during the time of alveolar development and is decreased after exposure of neonatal rats to a hyperoxia during this serious period. The VEGFR1 as well as VEGFR2 mRNA and protein were declined during exposure to a high-O_2_ condition. Expression of HIF-2α mRNA decreases in response to a hyperoxic O_2_, suggesting that the low levels of HIF-2α observed at high O_2_ concentrations are not stimulating VEGF expression. It is well known that this hyperoxic exposure protocol inhibits alveolarization in the rat pup (Boros et al., 1997; Manji et al., 2001); as a result, authors postulate that signaling *via* VEGF receptors may be a key mechanism for postnatal lung development and hyperoxic inhibition of this pathway may play a role to the observed decrease in septation of the alveoli (Hosford, 2003).

### Effects of Hyperoxia on Telomere Length and Cellular Senescence

The critical hallmarks of the aging process at the cell level include cell proliferation, telomere length shortening, and cellular senescence (Colloca et al., 2020). In aging individuals, the repeated HBOT interventions can increase PBMC telomere length by over 20%, with B cells displaying the highest change in telomere length (Hachmo and Hadanny, 2020). While several inborn and environmental factors are related with telomere shortening, the most common suggested mechanism is oxidative stress. Oxidative stress can occur from imbalances between the production of ROS and cellular scavengers. Telomeres are highly susceptible to oxidative DNA damage, which can induce telomere shortening and dysfunction (Barnes et al., 2019). The relation between telomere length shortening and oxidative stress has been debated for the past many years. Human cell culture studies constantly show that mild oxidative stress hastens telomere shortening, whereas antioxidants and free radical scavengers reduce shortening rates and increase the cellular proliferative lifespan (Von Zglinicki, 2002).

Many clinical studies on pathological conditions such as diabetes, inflammatory diseases, and Parkinson’s disease have demonstrated associations between oxidative stress markers, reactive oxygen species scavenger levels, and telomere length (Sampson et al., 2006). However, healthy participants did not show similar results (Reichert and Stier, 2017). Similarly, HBOT increased telomere length in hippocampus in rats. The number of senescent cells also decreased by 10%–37% after HBOT interventions, with T helper senescent cells being the most affected (Shwe et al., 2021). Likewise, HBOT was proposed as a non-pharmacological treatment, clinically accessible with well-known safety profile for senescent cell clearance (Hachmo and Hadanny, 2020).

### Side Effects of Hyperoxia

Oxygen toxicity involves the formation of reactive oxygen species that damage cell membranes and their components (Ikeda and Long, 1990). Oxygen free radicals are formed during normal oxidative metabolism in all aerobic cells and are reduced within the mitochondrial cristae, as part of normal oxidative phosphorylation (Boveris and Chance, 1973). The level of oxidant damage can be measured by a stoichiometric association between the rate of the formation of reactive oxygen species and rate of their elimination or quenching by antioxidants (Jamieson, 1989).

Although oxygen therapy is considered to be safe, at high dosage, it can be harmful and result in oxygen toxicity. Lengthy exposure to high oxygen pressure with a long disparity between ROS to scavengers can cause membrane lipid peroxidation and enzyme inhibition and modulations, most frequently seen in the central nervous system (CNS), that cause changes in neuronal metabolism and its associated electrical activity (Torbati et al., 1992). Lung is another organ that is comparatively susceptible to oxygen toxicity. Pulmonary oxygen toxicity can be manifested by chest tightness, cough, and a reversible decline of pulmonary function (Leach et al., 1998). Both CNS and pulmonary toxicity depend on the partial pressure of oxygen and the duration of exposure (Jain, 2017). Therefore, the current HBOT protocols comprise repeated daily sessions limited to 60–90 min with O_2_ partial pressure not beyond 2.4 ATA, as well as air brakes every 20–30 min. Using these new protocols, HBOT is considered to be safe; both pulmonary and CNS oxygen toxicity are very rare (Hadanny et al., 2016; Hadanny et al., 2019). Additionally, in patients without chronic lung diseases, the currently used HBOT protocols do not cause any pulmonary toxicity or changes in pulmonary functions following 60 repeated exposures (Hadanny et al., 2019).

In this review, we noticed that studies have reported varying degrees of toxicity of hyperoxia from no side effect to serious side effects depending on the dose and duration of oxygen exposure in the protocol of the studies. Some studies have shown that hyperoxia is a safe treatment option: Hyperoxia (100% O_2_) neither reproduces nor accelerates the pattern of alterations in glutathione redox state and PrSSG content observed during aging under normoxic conditions (Rebrin and Sohal, 2006). Upregulation of antioxidant, cytoprotective, and immediate early genes was observed in human endothelial cells following a hyperbaric oxygen exposure (100% O_2_ at 2.4 atm for 1 h) (Godman et al., 2010). These increases coincided with an improved resistance to a lethal oxidative stress. Administration of 80% O_2_ during colon surgery and 2 h following surgery did not deteriorate lung function or cause atelectasis (García-de-la-Asunción et al., 2011). Similarly, in severe TBI patients, exposure to hyperbaric O_2_ at 1.5 ATA for 60 min showed no increase in the CSF F2-isoprostane levels, microdialysate glycerol, and BAL inflammatory markers (IL-6 and IL-8), which were used to monitor potential O_2_ toxicity after exposure to HBOT (Rockswold et al., 2010).

On the other hand, other studies have shown that hyperoxia causes some serious side effects: HBOT protocol caused visual acuity changes, far sight acuity deterioration, and mild middle ear barotrauma (Amir et al., 2020). Similarly, increasing the exposure of the lens to hyperoxia could lead to mtDNA damage and increase the risk of nuclear cataract formation (Zhang et al., 2010). Therefore, lowering the levels of oxygen exposure around the lens might protect it against nuclear cataract formation by reducing the production of mtDNA damage induced by ROS. A remarkable initial pattern of mitochondria degeneration was also reported in *Drosophila* flight muscle under hyperoxia (100% O_2_) (Walker and Benzer, 2004). Exposure to elevated FiO_2_ during and after general anesthesia also showed unbalanced pro-oxidant/antioxidant equilibrium (Ottolenghi et al., 2019). The equilibrium of hyperoxia-exposed patients was unbalanced by high pro-oxidant and low anti-oxidant defense among patients undergoing elective abdominal surgery.

### Limitations

To the best of our knowledge, this is the first review to systematically investigate the current knowledge on the effects of short-term hyperoxia on aging biomarkers in preclinical and clinical models. However, the following limitations need to be considered in this systematic review. Systematic reviews are usually subject to publication bias of the studies showing no differences (Onishi and Furukawa, 2014). In addition, the quality assessment results of this review showed that six studies had high risk and two studies had unclear risk of random sequence generation bias. Similarly, one study had high risk and 7 studies had unclear risk of blinding of participants and personnel bias. Moreover, 8 studies had unclear risk of allocation concealment bias, blinding of outcome assessment bias, and blinding of outcome assessment bias (all case mortality), which could overrate their conclusion.

Furthermore, of the total studies in this review, 6 studies are non-randomized clinical trials and two studies are uncontrolled clinical trials. Although in human and animal experiments, variation between groups is limited by genetic homogeneity and standardized experimental conditions, lack of randomization and control can reduce the internal validity of the experiments (Bebarta et al., 2003), Moreover, most of the included studies did not show a power calculation and some studies used small sample size. Sample size calculation is required to reduce the incidence of false-negative or false-positive outcomes between groups and to keep the number of study participants used as low as possible considering the legal, ethical, and scientific requirements.

Variations in the study design including the use of animal, human, insects/worms, and cell line models; the use of different study participants in human models such as healthy individuals with different age-related diseases and different age groups; the use of different species of animals in animal models; and differences in hyperoxia exposure time, doses, and variation in outcome measures (biomarkers) hindered us to pool the data for meta-analysis and may also account for some of the conflicting results.

## Conclusion

This systematic review reveals that short-term hyperoxia treatment could be a feasible option to slow aging. Short-term hyperoxia treatment increased endogenous antioxidant enzymes that suppress ROS-associated cellular damage, significantly increased telomere length and clearance of senescent cells, and significantly upregulated or downregulated the expression of oxygen-sensitive transcription factors that are relevant to aging. This review also revealed that hyperoxia causes varying degrees of side effects from no side effect to serious adverse effects depending on the dose and duration of oxygen exposure. Some studies have demonstrated that hyperoxia neither reproduces nor accelerates the pattern of alterations in glutathione redox state and PrSSG content observed during aging. However, other studies have reported that increased exposure to hyperoxia causes some serious side effects such as visual acuity changes, far sight acuity deterioration, mild middle ear barotrauma, and mtDNA damage, and increases the risk of nuclear cataract formation. In general, it can be concluded that short-term hyperoxia causes positive dynamics of aging markers in both animal and human experiments. There is evidence of positive effects on certain parameters that reflect quality of life. That being said, there is no direct research to prove that short-term hyperoxia actually increases life expectancy in humans. This issue requires further study. Additional studies for better understanding the beneficial effects of short-term hyperoxia and for minimizing side effects are also necessary for optimal clinical application.

## Data Availability

The original contributions presented in the study are included in the article/Supplementary Material. Further inquiries can be directed to the corresponding author.

## Supplementary Material

Supplementary Table S1 The effect of hyperoxia on aging markers in human.

Supplementary Table S2 The effect of hyperoxia on aging markers in cell lines.

Supplementary Table S3 The effect of hyperoxia on aging markers in rats.

Supplementary Table S4 The effect of hyperoxia on aging markers in insects and worms.
