# Supplementary material for: Effects of Hyperoxia on Aging Biomarkers: A Systematic Review
Source: Front Aging. 2022 Jan 3;2:783144. doi: 10.3389/fragi.2021.783144 (PMC9261365; doi:10.3389/fragi.2021.783144)
Supplement: Supplementary file 1 [file DataSheet1.docx]

# 8 Supplementary Materials

**Supplementary Table S1:** The effect of hyperoxia on aging markers in human.

| Author , year | Country | Condition | Age in years | Number of cases with HBOT | Number of controls | Study design | hyperoxia protocol  (cases) | Protocol (controls) | Organ /tissue/ cells | Sample | Aging markers | Method for measurement | Results | Conclusions | Safety issues (Oxygen toxicity) |
| --- | --- | --- | --- | --- | --- | --- | --- | --- | --- | --- | --- | --- | --- | --- | --- |
| Amir et al., 2020 | Israel | Healthy | adults (>64) | 33 | 30 | RCT | 100% O2 at 2ATA for 90 min with 5-min air breaks every 20 min. | No intervention | Brain | head coils | CBF | MRI | Increased | HBOT induces cognitive enhancements in healthy aging adults via mechanisms involving regional changes in CBF. | Mild middle ear barotrauma, visual acuity changes, far sight acuity deterioration |
| Fratantonio et al., 2021 | Italy | Healthy | Adult (Mean, 21) | 12 | 0 | RCT | 30% O2 (MH) for 1 h | Baseline result | PBMCs | Blood | HIF-1α | Western Blotting | Activated | The return to normoxia after MH is sensed as a hypoxic trigger characterized by HIF-1 activation. On the contrary, HH and VHH induce a shift toward an oxidative stress response, characterized by NRF2 and NF- kB activation. | not reported |
|  |  |  |  |  |  |  |  |  |  |  | NRF2 | Western Blotting | Activated |  |  |
|  |  |  |  |  |  |  |  |  |  |  | NF- kB | Western Blotting | Not activated |  |  |
|  |  |  |  |  |  |  |  |  |  |  | GSH | reduced GSH Assay | Not activated |  |  |
|  |  |  |  |  |  |  |  |  |  |  | MMP-9 and MMP-2 | Zymographic Analysis | Activated (MMP-9) |  |  |
| Fratantonio et al 2021 | Italy | Healthy | Adult (mean, 21) | 12 | 0 | RCT | 100% O2 (HH) for 1 h | Baseline restult | PBMCs | Blood | HIF-1α | Western Blotting | Activated |  |  |
|  |  |  |  |  |  |  |  |  |  |  | NRF2 | Western Blotting | Activated |  |  |
|  |  |  |  |  |  |  |  |  |  |  | NF- kB | Western Blotting | Activated |  |  |
|  |  |  |  |  |  |  |  |  |  |  | GSH | GSH | Activated |  |  |
|  |  |  |  |  |  |  |  |  |  |  | MMP-9 and MMP-2 | Zymographic Analysis | Activated |  |  |
| Fratantonio et al 2021 | Italy | Healthy | Adult (mean, 21) | 12 | 0 | RCT | 140% O2 (VHH) for 1 h | Baseline restult | PBMCs | Blood | HIF-1α | Western Blotting | Not activated |  |  |
|  |  |  |  |  |  |  |  |  |  |  | NRF2 | Western Blotting | Activated |  |  |
|  |  |  |  |  |  |  |  |  |  |  | NF- kB | Western Blotting | Activated |  |  |
|  |  |  |  |  |  |  |  |  |  |  | GSH | reduced GSH Assay | Activated |  |  |
|  |  |  |  |  |  |  |  |  |  |  | MMP-9 and MMP-2 | Zymographic Analysis | Activated (MMP-9) |  |  |
| García-de-la-Asunción et al 2011 | Spain | Colon cancer | Adult (18-80) | 24 | 0 | RCT | 30% or 80% O2 throughout surgery | compared the two protocols | Colon | Colonic mucosa and blood | MDA levels | HPLC and spectrophotometry | lower in the 80% O2 group than in the 30% O2 group. | An increase in oxidative stress marker levels in blood and colonic mucosa occur when 30% O2 is used, possibly through an increase in XO enzymatic activity in the colonic mucosa. The 80% O2 prevented oxidative stress, with a reduction of lipid peroxidation and glutathione oxidation; this may be due to decreases in XO enzymatic activity and XO/(XO + XDH) ratio in the colonic mucosa. | Authors could not find respiratory complications in their  patients during the study period. Administration of  80% O2 during surgery and 2 hours after surgery  did not worsen pulmonary function or cause atelectasis. |
|  |  |  |  |  |  |  |  |  |  |  | GSSG | HPLC and spectrophotometry | lower in the 80% O2 group than in the 30% O2 group. |  |  |
|  |  |  |  |  |  |  |  |  |  |  | XDH | Fluorometric assays | XDH was higher, but XO/(XO + XDH) ratio was lower in the 80 % O2 group than in the 30% O2 group. |  |  |
|  |  |  |  |  |  |  |  |  |  |  | XO | Fluorometric assays | XO and XO/(XO + XDH) ratio were lower in the 80 % O2 group than in the 30% O2 group. |  |  |
| Hachmo et al 2020 | Israel | Healthy | Adults (> 64) | 35 | 0 | Uncontrolled trial | 100% O2 at 2ATA for 90 minutes with 5-minute air breaks every 20 minutes. | baseline restult | PBMCs | Blood | Telomere length | Flowcytometry analysis | Telomeres length of T helper, T cytotoxic, natural killer and B cells increased | The study indicates that HBOT may induce significant senolytic effects including significantly increasing telomere length and clearance of senescent cells in the aging populations | not reported |
|  |  |  |  |  |  |  |  |  |  |  | Senescent cells | Flowcytometry analysis | decrease in the number of senescent T helpers and T-cytotoxic senescent cells |  |  |
|  |  |  |  |  |  |  |  |  |  |  | HIF-1α | Flowcytometry analysis | HIF-1 α levels increased |  |  |
| Karu et al 2007 | Estonia | Coronary artery diseases | Adult (mean age >60) | 20 | 20 | RCT | >96% O2 for 120 min before cardioplegia. | 40% O2 for 120 min | Heart | Blood | troponin I | CIA | did not differ between the groups. | Exposure to >96% oxygen before cardioplegia did not attenuate Ischemia–reperfusion injury of the heart in patients undergoing coronary artery bypass grafting.The only potentially beneficial effect observed was the decreased transmyocardial release of interleukin-6. | not reported |
|  |  |  |  |  |  |  |  |  |  |  | CK-MB | CIA | did not differ between the groups. |  |  |
|  |  |  |  |  |  |  |  |  |  |  | lactate | photometry | did not differ between the groups. |  |  |
|  |  |  |  |  |  |  |  |  |  |  | GSH | Enzymatic method for GSH | More oxidised GSH was released in the hyperoxia group |  |  |
|  |  |  |  |  |  |  |  |  |  |  | Il-6 | SELIA | Decreased release of IL-6 |  |  |
| Karu et al 2015 | Estonia | coronary artery | Adult (>54) | 10 | 14 | RCT | 100 % O2 for 60 min. | 40% O2 for 60 min. | Heart | Myocardial tissue | MTRNR2L2 and MTRNR2L8 genes | RNA-seq analysis | MTRNR2L2 and MTRNR2L8 upregulated, and a "cell survival" network was activated . | Administration of100 % oxygen for 1 hour changes gene expression in the myocardium of the patients with coronary artery disease and may enhance cell survival capability. | not reported |
| Keramidas et al 2011 (78) | Slovenia | Healthy | Adult (mean age 25.5) | 5 | 5 | RCT* | 100% O2 for 2 h | 21 % O2 for 2 h | Kidney | Blood | EPO | SELIA | EPO concentration was significantly lower in Hyperbaric than in the Normobaric condition | The increased O2 tension suppresses the production of EPO in 3–5 h after the hyperoxic breathing intervention . | not reported |
| Ottolenghi et al 2019 | Italy | Abdominal surgery | Adult (18+) | 10 | 10 | RCT | 80% O2 during the surgery and until 2 h after the end of surgery | 40% O2 | Abdomain | blood | hydroperoxides | dROMs test | Hydroperoxides did not highlight any differences between the two groups nor within the same group, with respect to the baseline value. | MDA, the main end product of the peroxidation of polyunsaturated fatty acids directly influenced by O2, may represent the best marker to assess the pro-oxidant/antioxidant equilibrium after surgery. | unbalanced pro-oxidant/antioxidant equilibrium |
|  |  |  |  |  |  |  |  |  |  |  | antioxidants | Antioxidant barrier strength (OXYAdsorbent test) | antioxidant defense lower, in the 80% O2 group with respect to both the 40% O2 group and the baseline values . |  |  |
|  |  |  |  |  |  |  |  |  |  |  | NOx | Griess reaction | NOx was higher in the 80% O2 group than the 40% O2 group at 2 h after surgery. |  |  |
|  |  |  |  |  |  |  |  |  |  |  | MDA | TBARS test | The MDA concentration was higher 24 h after surgery in the 80% O2 group with respect to both the 40% O2 group and the baseline values . |  |  |
|  |  |  |  |  |  |  |  |  |  |  | HbSSG | MALDI-ToF | HbSSG in red blood cells was higher in the 80 % O2 group at the end of the surgery. |  |  |
| Rockswold et al 2010 | USA | severe traumatic brain injury | adult (Averege, 35) | 26 (HBO), and  21 (NBH) | 22 | RCT | 100% O2 for 60 min at 1.5 ATA (HBO2) or 100% O2 for 3 hours at 1.0 ATA (NBH) | Standard care without O2 intervention | Brain | Brain tissue, CSF, BAL | CBF | The nitrous oxide method | Hyperbaric O2 significantly increased CBF for 6 hours . | Hyperbaric O2 has a more robust posttreatment effect than NBH on oxidative cerebral metabolism. | No signs of pulmonary or cerebral O2 toxicity. |
|  |  |  |  |  |  |  |  |  |  |  | CSF lactate, glucose, pyru vate,& glycerol level, | an offline analyzer (CMA 600 microdial ysis analyzer). | CSF lactate concentrations decreased in both the HBO2 and NBH groups. The dialysate lactate levels in HBO2 decreased. Microdialysis lactate/pyruvate (L/P) ratios decreased in both HBO2 and NBH groups. No increase in microdialysate glycerol |  |  |
|  |  |  |  |  |  |  |  |  |  |  | CSF F2-isoprostane | enzyme immunoassay | No increase in the CSF F2- isoprostane levels |  |  |
|  |  |  |  |  |  |  |  |  |  |  | BAL fluid IL–8 and IL-6 | enzyme-linked immunosorbent assay | No increase BAL inflammatory markers, IL-6, and IL-8 |  |  |

MH= Medium hyperoxia; HH= High hyperoxia; VHH = Very high hyperoxia; RCT= randomized control trial; CBF= Cerebral blood flow; MRI =Magnetic Resonance Imaging; PBMCs = Peripheral blood mononuclear cells: HIF-1 α = Hypoxia inducible factor-1 α; NRF2= Nuclear factor (erythroid-derived 2)-like 2; NF- kB= Nuclear Factor kappa-light-chain-enhancer of activated B cells; GSH= Glutathione; MMP-9/2= Matrix metallopeptidase- 9 /2 ; MDA= Malondialdehyde; GSSG= oxidized glutathione; XDH= xanthine dehydrogenase; XO=xanthine oxidase; CK-MB= creatine kinase-MB; IL-6/8= interleukin-6/8; EPO = Erythropoietin; NOx = nitrates and nitrites; HbSSG= glutathionyl hemoglobin; MALDI-ToF= matrix-assisted laser desorption in a time-of-flight mass spectrometer; BAL = Bronchoalveolar lavage fluid; CSF= cerebro spinal fluid; CIA= chemiluminescent immunoassay; SELIA= sandwich enzyme linked immunoassay.

. **Supplementary Table S2:** The effect of hyperoxia on aging markers in cell lines.

| Author & year | Country | Cell line | Study design | hyperoxia protocol (for cases) | Protocol for controls | Aging markers | Method for markers measurement | Results | Conclusions | Safety issues (Oxygen toxicity) |
| --- | --- | --- | --- | --- | --- | --- | --- | --- | --- | --- |
| Godman et al 2010 | USA | HMEC-1 | non randemized CT | 100% O2 at 2.4 ATA for 1 h | no  intervention | Antioxidant gene expression in Nrf2, Integrin, and ERK/MAPK pathways. | Illumina microarray | The HSPA1A, HMOX1, and MT1X genes were upregulated, and collectively can provide protection from metabolic, proteotoxic, and oxidative forms of stress. ERK/MAPK signaling, including the activation of a number of immediate early genes can potentially influence apoptotic signaling. Endothelial cell viability in the HBO-treated cultures was significantly increased. | The data indicate that hyperbaric oxygen can induce protection against oxidative insults in endothelial cells and may provide an easily administered hormetic treatment to help promote healthy aging. | HBO is a relatively low-risk procedure that could be effectively applied as a broader preventative regimen to reduce the effects of aging |
| Pomatto et al 2019 | USA | MEFs | non randemized CT | 40% O2 for 2 weeks | 21% O2 for 2 weeks | Nrf2 signal transduction pathway | Western blotting | Hyperoxia increased baseline levels of Nrf2 and multiple transcriptional targets (20S Proteasome, Immunoproteasome, Lon protease, NQO1, and HO-1) | Changes the balance of Nrf2, Bach1, and c-Myc levels may account for dysregulation of stress responses and adaptive homeostasis during chronic hyperoxia and in ageing. | Not reported |
|  |  |  |  |  |  | Nrf2 inhibitors (Bach1 & c-Myc) | Western blotting | Bach1 and c-Myc, were strongly elevated by hyperoxia and appeared to exert a ceiling on Nrf2 signaling. Bach1 and c-Myc also increase during ageing and may thus be the mechanism by which adaptive homeostasis is compromised with age. |  |  |
|  |  |  |  |  |  | Cellular ability to adapt to signaling levels (1.0 μM) of H2O4 | Western blotting | Hyperoxia resulted in loss of cellular ability to adapt to signaling levels (1.0 μM) of H2O2. |  |  |

HMEC-1=Human microvascular endothelial cell line; MEFs =Mouse embryonic fibroblasts; HSPA1A= 70- kilodalton heat shock protein; HMOX1=heme oxygenase 1; MT1X= metallothionein 1X.

**Supplementary Table S3:** The effect of hyperoxia on aging markers in rats.

| Author & year | Country | Condition | Age | Number of cases with HBOT | Number of controls | Study design | hyperoxia protocol  (for cases) | Protocol for controls | Organ /tissue/ cells | Sample | Aging markers | Method for measurement | Results | Conclusions | Safety issues (Oxygen toxicity) |
| --- | --- | --- | --- | --- | --- | --- | --- | --- | --- | --- | --- | --- | --- | --- | --- |
| Hosford et al 2003 | Canada | Healthy | 4 -14 days | NM | NM | RCT | >95% O2 for 10 days | 21% O2 for 10 days | Lung | Lung tissue | VEGF | Real-Time Quantitative PCR | mRNA levels of VEGF increased in normoxic animals, but hyperoxia suppressed this increase. | hyperoxic exposure decreased VEGF levels, and decreased VEGF receptors (VEGFR1 and VEGFR2) levels. | not reported |
|  |  |  |  |  |  |  |  |  |  |  | VEGF receptors (VEGFR1 and EGFR2) | VEGF Immunoassay | VEGFR1 and VEGFR2 mRNA increased in normoxic animals, but they were decreased by hyperoxia |  |  |
|  |  |  |  |  |  |  |  |  |  |  | HIF-2 α | Western Immunoblotting | mRNA levels of HIF-2 α increased in normoxic animals, but hyperoxia suppressed this increase. |  |  |
| Shwe et al 2021 | Thailand | NA | > 20 weeks | 30 | 30 | non randemized CT | 100 % O2 at 2 ATA for 80 min /day for 14 days | atmospheric pressure (1ATA for 80min/day) for 14 days | Brain | Brain tissue, blood | Synaptic plasticity (Markers: LTD, LTP, dendritic spine density, expression of synaptic protein (PSD 95)) | Based on Pratchayasakul et al., 2011 protocol | Synaptic plasticity was restored/improved. | HBOT attenuated insulin resistance, cognitive impairment, hippocampal aging and pathologies. These findings suggest that HBOT restored insulin sensitivity, hippocampal functions, cognition in aging and aging-obese models. | not reported |
|  |  |  |  |  |  |  |  |  |  |  | hippocampal Insuline receptor function (marker: LTD) | Based on Pratchayasakul et al., 2011 protoocol | Iinsulin resuptor function was restored/improved |  |  |
|  |  |  |  |  |  |  |  |  |  |  | hippocampal ROS level | DCFHDA fluorescent dye | ROS was decreased |  |  |
|  |  |  |  |  |  |  |  |  |  |  | DCX | western blooting | could not restore neurogenesis |  |  |
|  |  |  |  |  |  |  |  |  |  |  | hippocampal autophagy (markers: p62 and LC3-II) | western blooting | Hyppocampal autophagy was restored |  |  |
|  |  |  |  |  |  |  |  |  |  |  | microglia hyperactivation | western blooting | microglial hyperactivation was attunated |  |  |
|  |  |  |  |  |  |  |  |  |  |  | hippocampal apoptosis | western blooting | hippocampal apoptosis reversed back to normal |  |  |
|  |  |  |  |  |  |  |  |  |  |  | Aging marker: beta-secretase (BACE1) | western blooting | BACE1 enzyme was reduced |  |  |
|  |  |  |  |  |  |  |  |  |  |  | Aging marker: telomere length | western blooting | telomere length was restored |  |  |
|  |  |  |  |  |  |  |  |  |  |  | Aging marker: SA-β-gal staining. | western blooting | The number of SA-β-gal positive cells was decreased |  |  |
| Zhang et al 2010 | China | Healthy | 2-14 months | 24 | 24 | non randemized CT | 60% O2 for 3 weeks | 21% O2 for 3 weeks | Eye | lens of the eyes | mtDNA damage | LX-PCR | Increased | These data emphasize the importance of DNA repair enzymes and antioxidant enzymes as targets to promote DNA repair and reduce production of ROS. | Increasing the exposure of the lens to  hyperoxia could lead to mtDNA damage and increase the risk of  nuclear cataract formation. |
|  |  |  |  |  |  |  |  |  |  |  | mtBER enzymes | Immunoblot analysis | Increased |  |  |
|  |  |  |  |  |  |  |  |  |  |  | 8-OHdG levels | Competitive ELISA | Increased |  |  |

NM= not mentioned; VEGF= Vascular Edndothelial Growth Factor; LTD =Insulin-induced long-term depression; LTP =long-term potentiation; SA-β-gal = senescence associated β falactosidase; DCFHDA =dichloro-hydrofluoresceindiacetate; DCX =neurogenesis; mtBER= mtDNA base excision repair; mtDNA= mitochondrial DNA; LX-PCR =Long extension polymerase chain reaction; 8-OHdG =8-hydroxy-20-deoxy-guanosine.

**Supplementary Table S4:** The effect of hyperoxia on aging markers in insects and worms.

| Author & year | Country | Insect / worm | Condition | Age | Number of cases with HBOT | Number of controls | Study design | hyperoxia protocol (for cases) | Protocol for controls | Organ /tissue/ cells | Sample | Aging markers | Method for measurement | Results | Conclusions | Safety issues (Oxygen toxicity) |
| --- | --- | --- | --- | --- | --- | --- | --- | --- | --- | --- | --- | --- | --- | --- | --- | --- |
| Rebrin et al 2006 | USA | Drosophila melanogaster | NA | 9-59 days | 12 groups | 12 groups | non randemized CT | 100% O2 from 10-day-old until death | 21 % O2 from 10-day-old until death | whole-body | whole-body homogenates | GSH | HPLC with electrochemical detection | hyperoxia had no marked effect on GSH concentration in both WT and YW flies | Results indicated that hyperoxia (100% oxygen) neither reproduces nor accelerates the pattern of alterations in glutathione redox state and PrSSG content observed during aging under normoxic conditions. | not reported |
|  |  |  |  |  |  |  |  |  |  |  |  | GSSG | HPLC with electrochemical detection | Under hyperoxia, YW flies did not exibit an increase in GSSG amount or a decline in GSH:GSSG ratio, whereas WT flies showed a decline in GSH:GSSG ratio only during the later part of hyperoxica. |  |  |
|  |  |  |  |  |  |  |  |  |  |  |  | PrSSG | HPLC with electrochemical detection | In neither strain there was a progressive increase in PrSSG amount under hyperoxia. |  |  |
| Walker et al 2004 | USA | Drosophila melanogaster | NA | 3-4 days old | 90 | 90 | non randemized CT | 100% O2 was passed through the box at a constant rate (300 ml/ min). | without intervention | Dorsal indirect flight muscle | Dorsal indirect flight muscle | Degeneration of mitochondria | Electron microscopy | In hyperoxia condition, mitochondrial degeneration occurs rapidly with in mitochondria of the flight muscle | Authors discovered a biomarker of oxidative damage to the mitochondria (swirls) within the flight muscle. Swirls may represent an early event in the deterioration of the mitochondrion. | degeneration of the mitochondria |
| Yanase et al 2008 | Japan | Caenorhabditis elegans | NA | 5-15 days | 100 | 100 | uncontrolled trial | 90% O2 for 3 hours per day for 10 days | mutant strains compared with wild strains | whole-body | whole-body homogenates | mitochondrial superoxide radical (O2-) levels | using O2- specific chemiluminesent probe, 2-methyl-6-p-methoxyphenylethynylimidazopyrazinone | The O2- levels in age 1 strain significuntly decreased after intermitent hyperoxia exposure. | These data suggest that oxidative stress induced hormesis is associated with a reduction in mitochondrial O2- production by activation of the antioxident system via Ins/IGF-1 signaling pathway | not reported |

NA= not applicable; GSH =Glutathione; GSSG= glutathione disulfide; PrSSG= protein mixed disulfides; O2-=superoxide; Ins/IGF-1= Insulin/Insulin-like growth factor-1: HPLC= High Performance Liquid Chromatography.
